# The Ethyl Acetate Extract of* Gynura formosana* Kitam. Leaves Inhibited Cervical Cancer Cell Proliferation via Induction of Autophagy

**DOI:** 10.1155/2018/4780612

**Published:** 2018-05-24

**Authors:** Jing Fan Ma, Peng Fei Wei, Chang Guo, Yuan Peng Shi, Yang Lv, Long Xin Qiu, Long Ping Wen

**Affiliations:** ^1^Department of Biological Science and Technology, School of Life Sciences, Longyan University, Longyan University, China; ^2^Key Laboratory of Preventive Veterinary Medicine and Biotechnology (Longyan University), Fujian Province University, Longyan, China; ^3^Fujian Provincial Key Laboratory for the Prevention and Control of Animal Infectious Diseases and Biotechnology, Longyan University, Longyan, Fujian 364012, China; ^4^Hefei National Laboratory for Physical Sciences at the Microscale, School of Life Sciences, University of Science and Technology of China, Hefei, Anhui 230027, China

## Abstract

*Gynura formosana* Kitam. belongs to the Compositae family and has been traditionally used for the prevention of cancer, diabetes, and inflammation in China. Previous studies had indicated that the ethyl acetate extract of* Gynura formosana* Kitam. leaves (EAEG) exhibited antioxidant and anti-inflammatory activity. In this report, we demonstrated that EAEG possessed potent anticancer activity through autophagy-mediated inhibition of cell proliferation. EAEG induced a strong cytostatic effect towards HeLa cells and, to a lesser extent, HepG2 and MCF-7 cells. This cytostatic effect of EAEG was not a consequence of increased apoptosis, as neither DNA fragmentation nor change in protein expression level for a number of apoptosis-related genes including Bid, Bax, Bcl-2, and caspase-3 was observed after EAEG treatment, and the apoptosis inhibitor Z-VAD-FMK did not inhibit the EAEG-elicited cytostatic effect. On the other hand, EAEG induced autophagy in a dose-dependent fashion, as shown by increased GFP puncta formation, enhanced conversion of the microtubule-associated protein light chain LC3-I to LC3-II, and downregulation of the p62 protein. Treating the HeLa cells with EAEG together with Chloroquine (CQ) further accelerated LC3 conversion and p62 clearance, indicating that EAEG induced complete autophagy flux. Importantly, the autophagy inhibitor 3-methyladenine (3MA) significantly abrogated the cytostatic effect of EAEG, strongly suggesting that EAEG inhibited HeLa cell proliferation through the induction of autophagy rather than apoptosis. Our results provided a novel and interesting mechanistic insight into the anticancer action of EAEG, supporting the traditional use of this plant for the treatment of the cancer.

## 1. Introduction

Cervical cancer is the second most common malignant tumor and the fourth main cause of cancer deaths in women worldwide [1, 2]. Over half a million cases are diagnosed every year, with the mortality rate of 9% in the world. In China, 130,000 cases are diagnosed with cervical cancer each year and account for 28% of new cases globally. The mortality rate is 14%, which is higher than that in other developing countries [3, 4]. Cervical cancer could be traditionally treated with surgery and chemoradiotherapy. However, anticancer drugs of such approach are highly toxic. Therefore, the development of new anticancer drugs with fewer side effects is very necessary. Plant-derived bioactive compounds are important resources for treating various forms of the cancer [5]. Numerous studies have demonstrated that plant extracts had antiproliferative or antitumor effects on tumor cells [6–8].

*Gynura formosana* Kitam. (family Compositae) is a frequently used vegetable in China. It exhibited various biological activities and had been used for the prevention of cancer, diabetes, and inflammation in the Chinese traditional system of medicine since ancient times. The leaves of* Gynura formosana* Kitam. contained several medicinally relevant components such as polyphenolics, flavonoids, alkaloids, terpenes, and saponins [9]. These substances might contribute to its biological activity. Our previous studies had exhibited that the ethyl acetate extract of* Gynura formosana* Kitam. leaves (EAEG) had high quantity of polyphenolics and flavonoids and had antioxidant and anti-inflammatory effects [9]. Recent studies showed that several bioactive compounds extracted from plants, mostly polyphenolics and flavonoids, played an important role in tumor cells inhibition effect [10, 11]. Distinctly, EAEG, a polyphenolic- and flavonoid-rich extract, is potentially relevant for preventing and treating tumor cells. However, the anticancer effect of EAEG was rarely reported. Therefore, in the present study, we investigated the effect of EAEG on the proliferation of human tumor cells and its underlying mechanism.

Autophagy, as a cellular self-digestive process, is also considered an evolutionarily conserved and lysosome-dependent process in all mammalian cells, which is associated with cell survival. It helps cells obtain nutrients under deprivation conditions. However, more and more studies [12] have shown that, despite its importance in maintaining cell homeostasis, autophagy plays also a vital role in the regulation of cell death. Actually, continuous “autophagy” will commit suicide. So autophagy may play a protective role against cancer

## 2. Materials and Methods

### 2.1. Reagents

All of cell culture reagents were purchased from HyClone Laboratories (Logan, UT, USA). Trehalose (Tre), Z-VAD-FMK, 3-methyladenine (3MA), and Chloroquine (CQ) were purchased from Sigma-Aldrich (St. Louis, MO, USA). LC3 antibody was purchased from Novus (Littleton, CO, USA) and glyceraldehyde phosphate dehydrogenase (GAPDH) antibody from Millipore. Bid, Bax, and Bcl-2 antibodies were all obtained from Santa Cruz Biotechnology (Santa Cruz, CA, USA). Caspase-3, p62, PCNA, and MCM7 antibodies were purchased from Cell Signaling Technology (Danvers, MA, USA). *β*-Actin antibody was purchased from Sigma Chemical Co. (St. Louis, MO, USA).

### 2.2. Preparation of EAEG

EAEG was prepared according to our previously published literature [9]. Briefly, 200 g of* Gynura formosana* Kitam. leaves powder was firstly soaked in petroleum ether for 24 h and then the extract was filtered, evaporated at 45°C, and dried in a freeze-dryer. The residue was extracted successively in chloroform and ethyl acetate in the same way. The yields of petroleum ether, chloroform, and ethyl acetate extracts were 9.9%, 2.3%, and 3.2%, respectively. In our previous studies, EAEG showed the strongest 1,1-diphenyl-2-picrylhydrazyl radical, 2,2-diphenyl-1-(2,4,6-trinitrophenyl)hydrazyl (DPPH) and 2,2′-azino-bis(3-ethylbenzthiazoline-6-sulfonic acid) (ABTS) radical scavenging activities in three extracts. The polyphenol and flavonoid contents in EAEG were 34.12 ± 0.01 mg/g gallic acid equivalent and 44.35 ± 0.02 mg/g catechin equivalent, respectively [9].

### 2.3. Cell Culture

HeLa and HepG2 cells were purchased from the American Type Culture Collection (ATCC, Manassas, VA, USA). MCF-7 cells were kindly provided by Professor Longping Wen (University of Science and Technology of China, Hefei, Anhui). HeLa cells stably expressing GFP-LC3 were constructed as previously described [13]. All cells were grown in DMEM supplemented with 10% FBS and 1% penicillin-streptomycin in a humidified atmosphere of 5% CO_2_ at 37°C.

### 2.4. Cell Viability Assay

Cell viability was determined by 3-(4,5-dimethylthiazol-2-yl)-2,5-diphenyltetrazolium bromide (MTT) assay according to the manufacturer's protocols (Sigma Chemical Co.). In brief, cells were cultured in 96-well flat bottom plates overnight and then treated with different concentrations (0, 15, 30, 45, 60, 75, 90, and 105 *μ*g/ml) of EAEG. After incubation for 24, 48, or 72 h, 100 *μ*l MTT solution (1 mg/ml) was added to each well and the plates were incubated at 37°C for 4 h. The supernatants were then removed and DMSO was added to each well to dissolve the MTT-formazan crystals formed by viable cells. Finally, the absorbance values were determined by a microplate reader (Bio-Rad, Hercules, CA, USA) at a wavelength of 570 nm.

### 2.5. Detection of DNA Fragmentation by Agarose Gel Electrophoresis

After treatment of HeLa cells with different concentrations (0, 45, 60, and 75 *μ*g/ml) of EAEG for 24 or 48 h, the phenol/chloroform/isoamyl alcohol was used to extract DNA from cells that had been digested with proteinase K. Cell lysate was collected, incubated at 55°C for 10 h, and then treated with 40 *μ*g/*μ*l DNase-free RNase at 37°C for 40 min. The DNA was precipitated with 4 volumes of ethanol and electrophoresed in 1.5% agarose gels and visualized by ethidium bromide staining using a Chemidox (BioRad, Hercules, CA).

### 2.6. Western Blot

HeLa cells were trypsinized, collected by centrifugation, and homogenized in ice-cold RIPA Lysis Buffer (Beyotime, Haimen, China). Protein concentration was analyzed by Bradford Protein Assay Kit (Beyotime, Haimen, China). 35 *μ*g of protein sample was loaded and separated on an SDS/PAGE gel and transferred to PVDF membrane (Millipore, Billerica, MA, USA). The membrane was incubated with Tris-buffered saline containing 0.1% Tween-20 and 5% nonfat dry milk followed by 2 h incubation at the room temperature with antibody to Bax, Bid, Bcl-2, caspase-3, *β*-actin, GAPDH, p62, and LC3, respectively, and then with horseradish peroxidase-conjugated anti-mouse or anti-goat or anti-rabbit IgG (Sigma, 1 : 2000). Detection was achieved using enhanced chemiluminescence (ECL) kit (Beyotime, Haimen, China).

### 2.7. Observation of GFP-LC3 Puncta

HeLa cells stably expressing GFP-LC3 were incubated in 24-well plates and treated with EAEG for 24 h. GFP-LC3 puncta were observed under fluorescent microscopy (Olympus IX71, Tokyo, Japan). All of photos were captured randomly.

### 2.8. Statistical Analysis

All of the results were expressed as mean ± standard error of mean (SEM). Statistical significances were analyzed by one-way analysis of variance (ANOVA) with Dunnett's multiple comparison tests (SPSS version 22.0 software, SPSS Inc., Chicago, IL, USA). *p* < 0.05 was considered statistically significant.

## 3. Results

### 3.1. EAEG Exhibited Cytostatic Effect in Tumor Cells

**Figure 1:**
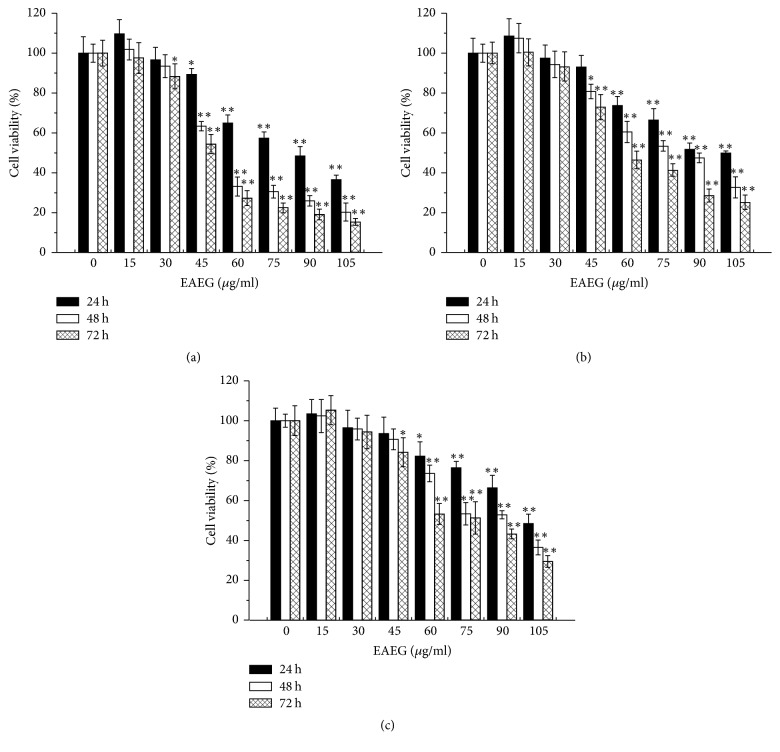
Cell viability comparison of HeLa (a), HepG2 (b), and MCF-7 (c) cells treated with different concentrations of EAEG (0–105 *μ*g/ml) for 24, 48, and 72 h. The data were expressed as mean ± SEM (*n* = 6). ^*∗*^*p* < 0.05 and ^*∗∗*^*p* < 0.01 with respect to the control group. EAEG: ethyl acetate extract of* Gynura formosana* Kitam. leaves.

To investigate the cytostatic effect of EAEG on the tumor cells, HeLa cells (cervical cancer), HepG2 (liver cancer), and MCF-7 cells (breast cancer) were exposed to various concentrations of EAEG for up to 3 days in vitro. As shown in Figure 1, EAEG within a dosage range of 0–105 *μ*g/ml decreased the cell viability in a time- and dose-dependent manner in all three cell lines. Significant (*p* < 0.05) viability-reducing effect was observed at EAEG concentrations of 30 *μ*g/ml and the time of 72 h in HeLa cells (Figure 1(a)). In comparison, significant decrease of cell viability in HepG2 and MCF-7 cells started at EAEG concentration of 45 *μ*g/ml at the time of 48 h and 72 h, respectively (Figures 1(b) and 1(c)). Moreover, the IC_50_ value of EAEG for HeLa, HepG2, and MCF-7 cells was 81.47, 100.94, and 104.76 *μ*g/ml at 24 h, respectively (Figure 1). As HeLa cells possessed the lowest IC_50_ value, they were selected for subsequent studies.

### 3.2. The Cytostatic Effect of EAEG Was Not due to Enhanced Apoptosis

**Figure 2:**
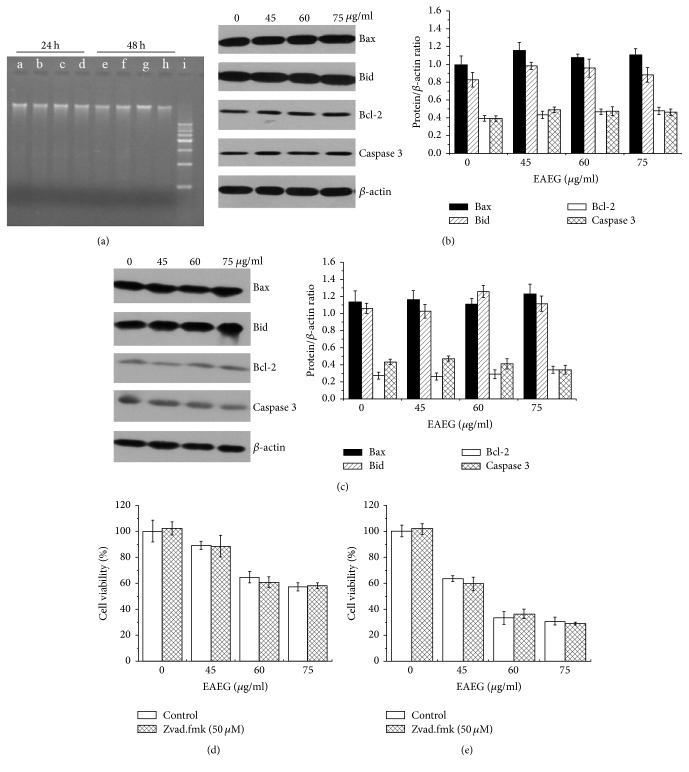
EAEG treatment did not induce apoptotic cell death in HeLa cells. (a) DNA fragmentation analysis. Lines a and e, controls treated for 24 and 48 h, respectively; lines b and f, 45 *μ*g/ml EAEG treated for 24 and 48 h, respectively; lines c and g, 60 *μ*g/ml EAEG treated for 24 and 48 h, respectively; lines d and h, 75 *μ*g/ml EAEG treated for 24 and 48 h, respectively; line i, DNA marker. ((b) and (c)) The expression of Bid, Bax, Bcl-2, and caspase-3 in HeLa cells treated with different concentrations of EAEG (0, 45, 60, and 75 *μ*g/ml) for 24 h (b) or 48 h (c). *β*-Actin served as the loading control. The relative protein/*β*-actin ratios were calculated using ImageJ. ((d) and (e)) Cell viability of HeLa cells treated with/without Z-VAD-FMK (50 *μ*M) and different concentrations of EAEG for 24 h (d) or 48 h (e). The data were expressed as mean ± SEM (*n* = 3). EAEG: ethyl acetate extract of* Gynura formosana* Kitam. leaves.

The most likely explanation for the observed cytostatic effect of EAEG on tumor cells was the induction of apoptotic cell death by EAEG. To investigate this possibility, we conducted DNA fragmentation (the hallmark of apoptotic cell death) assay and also assessed the expression level for a number of apoptosis-related proteins. Treatment of HeLa cells with EAEG at a range of concentrations up to 75 *μ*g/ml for either 24 h or 48 h induced no DNA fragmentation (Figure 2(a)) and did not alter the expression level of Bid, Bax, Bcl-2, and caspase-3 (Figures 2(b) and 2(c)). Furthermore, Z-VAD-FMK, an apoptosis inhibitor [14], did not affect the viability-reducing effect of EAEG (Figures 2(d) and 2(e)). Taken together, these results indicated that the cytostatic effect of EAEG in HeLa cells was apoptosis-independent.

### 3.3. EAEG Induced Complete Autophagy

**Figure 3:**
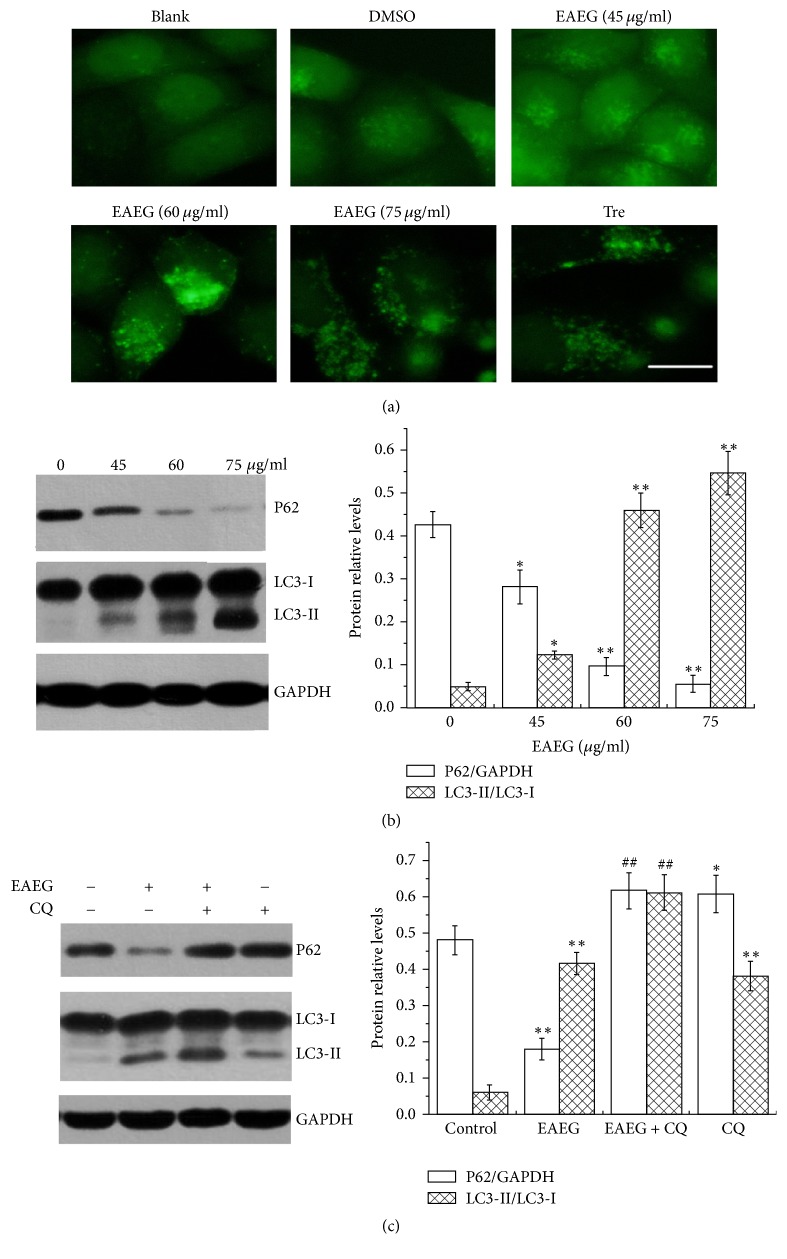
EAEG induced autophagy in HeLa cells. (a) Analysis of GFP-LC3 puncta formation in GFP-LC3/HeLa cells under fluorescence microscopy. Cells were treated for 24 h with DMEM only (blank), EAEG solvent (DMSO) as control, different concentrations (45, 60, and 75 *μ*g/ml) of EAEG, or the positive control trehalose (Tre) (scale bar = 20 *μ*m.). (b) The expression analysis of the autophagy-related proteins p62 and LC3 by Western blot. HeLa cells were treated for 24 h with different concentrations (0, 45, 60, and 75 *μ*g/ml) of EAEG. (c) The relative protein levels of p62 and LC3-II in HeLa cells treated with 60 *μ*g/ml EAEG with or without CQ (50 mM) for 24 h. GAPDH served as the loading control. The protein relative ratio was calculated using ImageJ. The data were expressed as mean ± SEM (*n* = 3). ^*∗*^*p* < 0.05 and ^*∗∗*^*p* < 0.01 with respect to the control group. ^##^*p* < 0.01 with respect to EAEG treatment group. EAEG: ethyl acetate extract of* Gynura formosana* Kitam. leaves.

**Figure 4:**
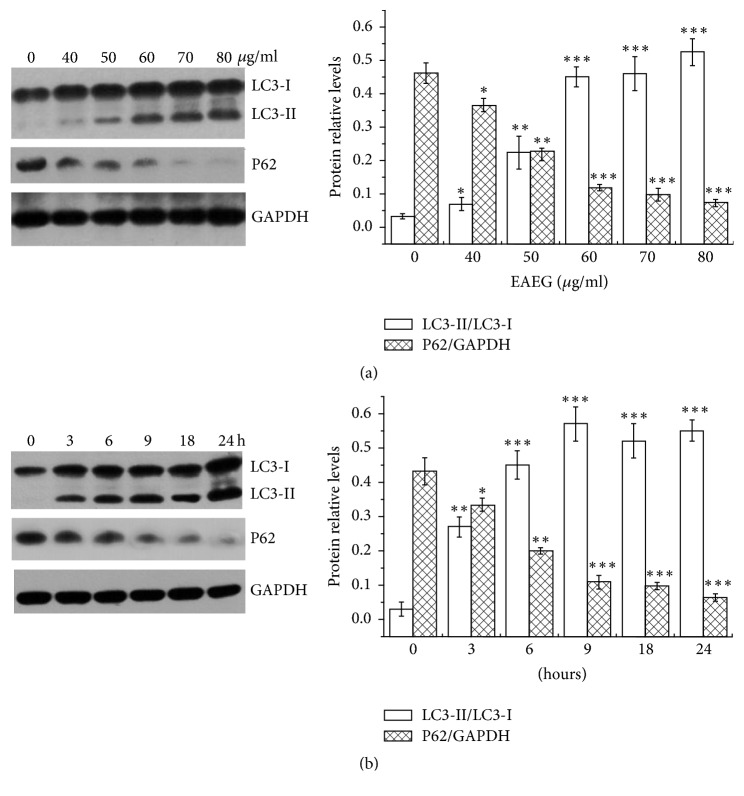
The conversation of LC3-I to LC3-II. Protein level of p62 was measured by Western blot. (a) HeLa cells were treated with EAEG at a concentration of 0, 40, 50, 60, 70, or 80 *μ*g/ml for 24 h. (b) HeLa cells were treated with 60 *μ*g/ml EAEG for 0, 3, 6, 9, 18, or 24 h. GAPDH served as the loading control. The relative protein ratio was calculated using ImageJ. The data were expressed as mean ± SEM (*n* = 3). ^*∗*^*p* < 0.05, ^*∗∗*^*p* < 0.01, and ^*∗∗∗*^*p* < 0.001 with respect to the control group. EAEG: ethyl acetate extract of* Gynura formosana* Kitam. leaves.

Autophagy modulation has been demonstrated to be a critical mediator of anticancer efficacy for many drugs [15–17]. Our results above showed that EAEG did not cause apoptotic cell death in HeLa cells, so we next investigated whether EAEG induced autophagy. We used GFP-LC3/HeLa, a cell line stably expressing GFP-LC3, a fusion protein between green fluorescent protein (GFP) and microtubule-associated light chain 3 (LC3), protein. GFP-LC3 protein accumulated to form green fluorescence puncta on autophagosome membranes upon autophagy [13]. Similar to trehalose, the positive control, EAEG at the various concentrations induced significant puncta formation, while DMSO (EAEG solvent) did not, indicating that EAEG elicited autophagy (Figure 3(a)). In addition, EAEG treatment significantly enhanced the conversion of LC3-I to LC3-II and reduced the protein level of p62, a common autophagic substrate, with significant (*p* < 0.01) changes observed at 60 *μ*g/ml and above for 24 h (Figure 4(a)), lending further proof for the occurrence of autophagy and also suggesting that the autophagy induced by EAEG was complete with normal cargo degradation (Figure 3(b)). To further support the latter conclusion, we treated HeLa cells with 60 *μ*g/ml EAEG for 24 h in the presence or absence of Chloroquine (CQ), a classical later-stage autophagy inhibitor. Earlier literature showed that the degradation of LC3-II would be impaired in cells treated with saturating dose of CQ (50 mM) [18]. Indeed, a further increase in the level of LC3-II was seen in HeLa cells treated with EAEG plus CQ as compared to HeLa cells treated with EAEG alone (Figure 3(c)), strongly suggesting that EAEG induced complete autophagy with normal flux. As would be expected, p62 degradation was retarded by CQ (Figure 3(c)).

### 3.4. EAEG Induced Dose- and Time-Dependent Autophagy

To further characterize the autophagic response elicited by EAEG, we performed dose- and time-dependent studies, using LC3-I/LC3-II conversion and p62 protein level as the measures. HeLa cells were treated with different concentrations (0, 40, 50, 60, 70, and 80 *μ*g/ml) of EAEG for 24 h or with 60 *μ*g/ml EAEG for different times (0, 3, 6, 9, 18, and 24 h). A dose-dependent conversion of LC3-I to LC3-II, accompanied by a corresponding decrease in the level of p62, was observed following EAEG treatment in HeLa cells, with significant changes observed at 40 *μ*g/ml and above (Figure 4(a)). The conversion of LC3-I to LC3-II and the corresponding decrease in the level of p62 were also time-dependent, with significant change observed at 3 h and beyond, following EAEG treatment in HeLa cells (Figure 4(b)).

### 3.5. The Autophagy Induced by EAEG Caused HeLa Cells Death

**Figure 5:**
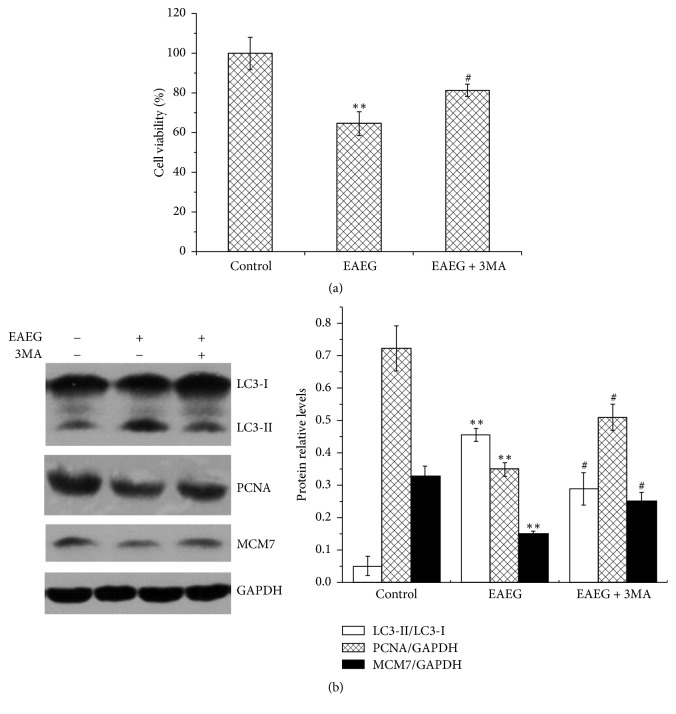
EAEG induced HeLa cells undergoing autophagic cell death. (a) Cell viability of HeLa cells treated with 60 *μ*g/ml EAEG for 24 h in presence or absence of 5 mM 3MA. (b) Western blot analysis of autophagy and proliferation proteins. GAPDH served as the loading control. The protein relative ratio was calculated using ImageJ. The data were expressed as mean ± SEM (*n* = 3). ^*∗∗*^*p* < 0.01 with respect to the control group and ^#^*p* < 0.05 with respect to EAEG treatment group. EAEG: ethyl acetate extract of* Gynura formosana* Kitam. leaves.

Did the autophagy induction contribute to the cytostatic effect elicited by EAEG? To assess this possibility, we measured cell viability after the treatment of HeLa cells with EAEG plus 3MA, a class III-PI3K autophagy inhibitor. As shown in Figure 5(a), 60 *μ*g/ml EAEG treatment for 24 h reduced the cell viability to 64.68%, and this effect was partially offset by the cotreatment with 5 mM 3MA (Figure 5(a)), which inhibited significantly the autophagy induced by EAEG (Figure 5(b)). Furthermore, EAEG significantly decreased the level of PCNA and MCM7, two widely used cell proliferation markers [19], and this effect was also partially abrogated by 3MA (Figure 5(b)). We thus concluded that EAEG elicited a cytostatic effect on cancer cells through blocking autophagy-mediated cell proliferation rather than inducing apoptosis.

## 4. Discussion

*Gynura formosana* Kitam. had been used traditionally for the prevention of cancer, diabetes, and inflammation in China. Our previous results showed that EAEG contained polyphenols and flavonoids and had antioxidant and anti-inflammatory activities [9]. These collective results suggested that EAEG might present antitumor activity. However, there was little evidence showing the antitumor effects of EAEG.

The plant-derived compounds had served as a main source of drugs, and about more than 50% of pharmaceuticals were derived from natural compounds [20]. To determine if EAEG could affect the cells proliferation, we first investigated the cytostatic activity of EAEG on HeLa, HepG2, and MCF-7 cells. Our results showed that EAEG reduced the cell viability in time- and dose-dependent manners in three cell lines and was more toxic to HeLa cells (Figure 1), which suggested that EAEG might have better chemotherapeutic potential to cervical cancer. To elucidate the possible mechanism involved in the above cytostatic effects, we assumed that HeLa cells would undergo apoptotic cell death after EAEG treatment. However, any DNA fragmentation could not been detected in HeLa cells treated with even 75 *μ*g/ml EAEG for 48 h. We further investigated the proapoptosis, antiapoptosis, and apoptosis-related proteins expression in HeLa cells treated with EAEG. Bax and Bid were both proapoptotic proteins and could promote apoptotic proteins release. However, the antiapoptotic protein Bcl-2 induced by various pathologic and physiologic stimuli could suppress apoptotic proteins efflux [21–23]. Caspase was a sensitive indicator of apoptosis [24]. We evidenced, in this study, that EAEG could not induce the activation of Bax, Bid, Bcl-2, and caspase-3 in HeLa cells by Western blot analysis, suggesting that apoptotic pathway was not triggered by EAEG treatment. Most importantly, the apoptosis inhibitor Z-VAD-FMK did not affect the viability-reducing effect of EAEG (Figures 2(d) and 2(e)). This finding served as a convincing evidence for nonapoptotic cell death in EAEG treated HeLa cells.

Autophagy was considered to be main way to inhibit tumor cells proliferation when cells underwent nonapoptotic cell death [25, 26]. Increasing evidences had suggested that autophagy could be induced by many natural products in various anticancer therapies. Triterpenoid B-group soyasaponins could induce autophagy in colon cancer cells by inhibition of Akt signaling and enhanced ERK1/2 activity [27]. Polyphenols extracts could induce autophagy, which was advantageous to inhibit neurodegeneration and cancer process [28]. Recent studies demonstrated that* Emblica officinalis* extracts could also induce autophagy and inhibit human ovarian cancer cell proliferation [29]. These reports supported us to hypothesize that HeLa cells death caused by EAEG could involve autophagy program. Autophagy was a physiologically and pathologically regulated process and played an important role in the maintenance of intracellular homeostasis in every eukaryotic cell [30–32]. It started with inclosing cytoplasmic contents in a vacuole called autophagosome and then autophagosome fused with lysosome to form autolysosome, where substances inside could be degraded and recycled [33]. Autophagy could be initiated by some autophagy-related proteins such as the microtubule-associated protein LC3, which was a reliable autophagic marker [34]. LC3 was a constitutively expressed protein in mammalian cells and could be processed to form cytosolic LC3-I. During autophagy, LC3-I was recruited to the autophagosome, where LC3-II was generated by fusing LC3-I with phosphatidylethanolamine, and then cellular autophagosome puncta including LC3-II were formed [35]. Our results demonstrated that EAEG upregulated GFP puncta formation (Figure 3(a)) and increased the conversion of LC3-I to LC3-II in HeLa cells (Figure 3(b)), suggesting the recruitment of LC3-II upon EAEG administration. Furthermore, we analyzed the protein level of p62, another autophagic marker [36], to study induction of autophagy. p62 could be trapped by LC3, incorporated into the autophagosome, and then degraded via autophagy. Correspondingly, the protein level of p62 should be decreased in the cells because of autophagy. Our results showed that the protein level of p62 reduced significantly in a dosage-dependent manner after EAEG treatment (Figure 3(b)), indicating an apparent induction of autophagy. However, these results could not explain if EAEG could induce the complete autophagy flux. For instance, calcium phosphate precipitates (CPP) induced both autophagosome synthesis and reduction of autophagy flux due to impaired autophagosome-lysosome fusion [37]. Therefore, it was very necessary to evaluate if the autophagy induced by EAEG was complete autophagy flux including autophagosome formation and autophagosome-lysosome fusion. Our results showed that the protein levels of both LC3-II and p62 strongly increased in HeLa cells treated with EAEG together with CQ, compared to cells treated with EAEG alone (Figure 3(c)). These results confirmed that EAEG could induce complete autophagy flux.

To confirm autophagy intensity, we estimated the conversion of LC3-I to LC3-II and protein level of p62. EAEG elicited dose- and time-dependent autophagic induction in HeLa cells through the conversion of LC3-I into LC3-II and decrease of protein level of p62 (Figure 4), strongly suggesting that EAEG had autophagic activity. Additionally, we also demonstrated that 3MA could partially abrogate EAEG induced HeLa cells death, the conversion of LC3-I to LC3-II, and decreasing level of PCNA and MCM7. The presented collective results indicated that EAEG might inhibit HeLa cells proliferation by inducing autophagy.

In summary, our studies indicated that EAEG, a natural plant extract, could be a potentially effective therapeutic agent for treating cervical cancer. Our experimental evidences also supported the traditional use of this plant for the treatment of the deadly disease cancer. The identification of such biological compounds in this plant and the mechanism against tumor cells are our research targets in the future.
